# Impact of Treatment with Direct Acting Antiviral Drugs on Glycemic Control in Patients with Hepatitis C and Diabetes Mellitus

**DOI:** 10.1155/2020/6438753

**Published:** 2020-01-13

**Authors:** Pradeep Kumar Mada, Matthew E. Malus, Arvin Parvathaneni, Bing Chen, Gabriel Castano, Sharon Adley, Maureen Moore, Michinari Hieda, Mohammed J. Alam, Mark Feldman, John William King

**Affiliations:** ^1^Internal Medicine Department, Texas Health Presbyterian Hospital, Dallas, TX, USA; ^2^Infectious Diseases, Louisiana State University Health Sciences Center-Shreveport, Shreveport, LA, USA; ^3^Internal Medicine, Louisiana State University Health Sciences Center-Shreveport, Shreveport, LA, USA; ^4^Neurology, Louisiana State University Health Sciences Center-Shreveport, Shreveport, LA, USA; ^5^Pediatrics, UT Health Sciences Center, San Antonio, USA; ^6^Institute for Exercise and Environmental Medicine, University of Texas Southwestern Medical Center, USA

## Abstract

**Aim:**

To assess the effect of treating chronic hepatitis C virus (HCV) infection with direct acting antiviral drugs (DAAs) on glycemic control in patients with concomitant diabetes mellitus (DM).

**Methods:**

We performed a retrospective case-control study in a viral hepatitis ambulatory clinic in Shreveport, Louisiana, during the period 11/01/2014 to 12/31/2017. All the clinic patient ages 18 years and above with treatment-naïve/biopsy-proven chronic hepatitis C and DM (hemoglobin A1C level ≥ 6.5%) who were eligible for treatment were included in the study. Of 118 such patients, 59 were treated with oral DAAs for 8-12 weeks with the goal of achieving a sustained virologic response (SVR). A control group of 59 patients did not receive treatment for their hepatitis C and was followed in the clinic. Patients in the control group did not receive treatment either due to insurance issues or refusal of hepatitis C treatment.

**Results:**

Fifty-five of the 59 patients treated with DAAs (93%) achieved a SVR. Six months after treatment completion, their mean ± SEM HbA1C level had decreased by 1.1 ± 0.03% (*P* < 0.0001). Four of the 59 patients treated with DAAs did not achieve a SVR. Their mean HbA1C 6 months after treatment completion had increased by 0.8 ± 0.2%. Furthermore, there was no improvement in HbA1C levels over time in the untreated group (mean HbA1C increase, 0.2 ± 0.05%; *P* < 0.0001 vs. the treatment group, which had a mean HbA1C decrease of 0.9 ± 0.2%).

**Conclusion:**

This controlled study demonstrated that treatment of chronic hepatitis C with DAAs results in statistically significant and meaningful reductions in hemoglobin A1C levels in patients with coexisting diabetic mellitus if a SVR is achieved.

## 1. Introduction

Diabetes mellitus (DM) is strongly associated with chronic hepatitis C virus (HCV) infection. It has been estimated that more than 30% of patients with chronic HCV infection have blood glucose abnormalities, specifically impaired fasting glucose or overt DM [1]. Cohort studies suggest that successful treatment of chronic HCV infection with currently available direct acting antiviral agents (DAAs) improves glycemic control in these diabetics [2–11]. We performed a retrospective study to assess the effect of a sustained viral response (SVR) to chronic HCV treatment with DAAs on glycemic control in a cohort of HCV patients with overt DM seen at a viral hepatitis clinic in Shreveport, Louisiana. An untreated group of patients with chronic hepatitis C was included as controls.

## 2. Methods

### 2.1. Study Population

All adult patients (age ≥ 18 years) with treatment-naïve, biopsy-proven chronic hepatitis C and concomitant DM (hemoglobin A1C (HbA1C) level ≥ 6.5% [12]) seen in a viral hepatitis clinic in Shreveport, Louisiana, clinic between November 1, 2014, and December 31, 2017, were eligible for inclusion in the study. Clinic patients with chronic hepatitis C and with DM were identified by ICD-10-CM codes B18.2 and E11.9, respectively. We included a total of 118 HCV-infected diabetic patients (51 men and 67 women), 59 of whom were subsequently treated by their clinic physicians with DAAs and 59 controls who were not treated for hepatitis C (because of insurance or refusal of hepatitis C treatment) and were followed in the clinic. Clinic patients treated with ribavirin or PEGylated interferon-based regimens were excluded from the study. All patients were screened for hepatocellular carcinoma (HCC), and none were found to have HCC. We recorded baseline characteristics including age, gender, and race. We also recorded the fibrosis stage using the METAVIR system on liver biopsy which was done before one month of treatment initiation [13, 14]; HbA1C, body mass index (BMI), and the list of diabetic medication(s) with doses were recorded within one month pretreatment (baseline/index) and six months postcompletion of HCV treatment in the treatment group, and in the untreated group, the above characteristics were measured at the time of liver biopsy (baseline/index) and then nine months after liver biopsy. Hep C RNA was checked at 4, 8, and 12 weeks during hepatitis C treatment and at 6 months after completion of antiviral therapy. SVR12 was considered as a cure marker.

In the SVR group, 6 patients were on insulin, 9 patients were on oral antidiabetic medication, 25 patients were on both insulin and oral antidiabetic medication, and 15 patients were on diet modification. All four in the non-SVR group were on oral antidiabetics. In the control group, 19 patients were on insulin, 33 were on oral antidiabetic medication, and 7 patients were on both insulin and oral antidiabetics. There was no change in antidiabetic medications and dosage during the study period. The two groups of patients are compared in Table 1. Patients treated with DAAs were, on the average, 5 years older than untreated controls (*P* < 0.0001 by group *t*-test). There were no significant differences in baseline HbA1C levels, gender and racial distributions, or baseline body mass index (BMI). Likewise, there were no significant differences in baseline viral loads and in serum aminotransferase levels. Whereas most of the patients in both groups had HCV genotype 1 (Table 1), there was a significant difference in genotype distribution between treated and untreated patients (genotype 1 vs. non-1, *P* = 0.034).

Liver biopsy tissue samples were analyzed by a senior pathologist. The degree of hepatic inflammation was graded as A0 (no inflammation), A1 (mild inflammation), A2 (moderate inflammation), and A3 (severe chronic hepatitis). Fibrosis was graded as F0 (no fibrosis), F1 (mild fibrosis), F2 (significant fibrosis), F3 (severe fibrosis), and F4 (cirrhosis). The METAVIR scoring system was used to assess the extent of hepatic inflammation and fibrosis. The activity score was graded based on the intensity of necroinflammatory lesions (A0 = no activity, A1 = mild activity, A2 = moderate activity, and A3 = severe activity), and the fibrosis score was assessed on a five-point scale (F0 = no fibrosis, F1 = portal fibrosis without septa, F2 = few septa, F3 = numerous septa without cirrhosis, and F4 = cirrhosis) [13, 14].

There were no significant differences in the degree of baseline hepatic inflammation or fibrosis in treated versus untreated patients (Table 1). Sofosbuvir-based therapy was used to treat HCV infection in 51 of the 59 treated patients. Seven of the 8 remaining patients received Zepatier (elbasvir/grazoprevir); the other received a Viekira Pak (ombitasvir, paritaprevir, and ritonavir; dasabuvir). Patients were treated for 8 to 12 weeks according to AASLD-IDSA guidelines [15]. SVR was defined as undetectable HCV in the blood 12 weeks or more after completing treatment.

The study was approved by an Institutional Review Board on August 23, 2017.

### 2.2. Statistical Analyses

Statistical analyses were carried out using the IBM SPSS version 20 software program (IBM® SPSS® Statistics, Armonk, NY). We performed *t*-tests to determine if there were significant differences between mean results of continuous variates. For categorical variates, Fisher exact tests were used. We also performed a multiple logistic regression analysis to determine to what extent covariates independently affected blood HbA1C levels. Two-sided *P* values < 0.05 were considered significant.

## 3. Results

Of the 59 patients treated with DAAs, 55 achieved a SVR (93%). The four patients not achieving a SVR with treatment had HCV genotype 1 (*n* = 3) or 2 (*n* = 1).

**Figure 1:**
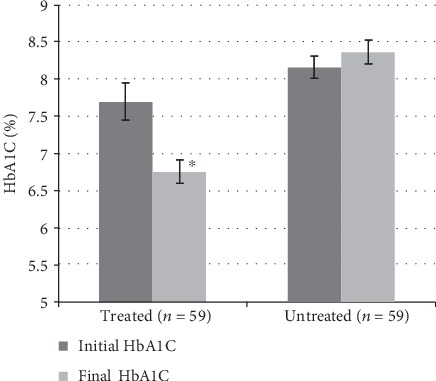
Mean ± SEM initial and final hemoglobin A1C levels (%) in treated patients (*n* = 59) and untreated patients (*n* = 59). The difference between the final HbA1C level in treated and untreated patients was significant, as was the difference between the initial and final HbA1C levels in treated patients (^∗^*P* < 0.0001).

As shown in Figure 1, mean ± SEM HbA1C levels decreased over time in the treatment group, falling from 7.7 ± 0.3% to 6.8 ± 0.2% (*P* < 0.0001 by paired *t*-test). In contrast, the mean HbA1C level in the untreated group increased slightly from 8.2 ± 0.2% to 8.4 ± 0.1% over the same time frame. The mean posttreatment HbA1C level in the treatment group was significantly lower than the final HbA1C level in the untreated group (*P* < 0.0001 by group *t*-test). Likewise, the mean ± SEM change in the blood HbA1C level from the baseline to study completion was −0.9 ± 0.2% in treated patients and +0.2 ± 0.05% in untreated patients (*P* < 0.0001 by group *t*-test).

**Figure 2:**
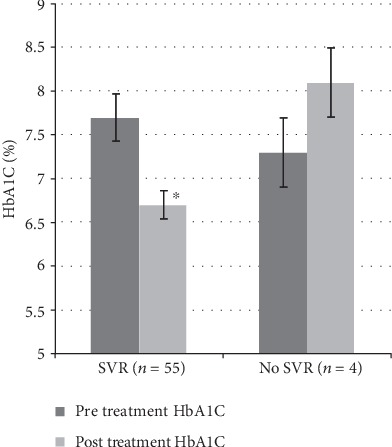
Mean ± SEM initial and final hemoglobin A1C levels (%) in the 59 treated patients shown in Figure 1, subgrouped as to whether patients had achieved a SVR (*n* = 55) or no SVR (*n* = 4). The difference between the SVR and no SVR patients was significant, as was the difference between pretreatment and posttreatment HbA1C levels in SVR patients (^∗^*P* < 0.0001). SVR: sustained virologic response.

As shown in Figure 2, the mean HbA1C decreased by 0.9% from 7.7 ± 0.7% to 6.7 ± 0.6% in the subgroup of 55 treated patients who achieved a SVR (*P* < 0.0001 by paired *t*-test). Forty-three of these patients had a decrease in HbA1C (31 patients had a greater than 0.5% reduction), 2 had no change, and 10 had an increase in their HbA1C level of 0.1 to 1.5%. The largest individual HbA1C decrement was from 13.6% to 8.1%. All 4 treated patients who did not achieve a SVR had an increase in their HbA1C level, averaging 0.8 ± 0.3% (Figure 2). The largest individual HbA1C increment in these 4 patients was from 7.1% to 8.2%.

There were no significant difference in the baseline versus the final mean BMI in either group of patients and no significant differences between the two groups. The BMI changed by −0.3 ± 0.3 and 0 ± 0.1 kg/m^2^ in treated and untreated patients, respectively (*P* = 0.50). By univariate analysis, none of the following was associated with a significant improvement in blood HbA1C levels in the 118 patients over time: age, gender, race, baseline BMI, viral load, HCV genotype, baseline serum ALT or AST, and baseline hepatic inflammation or fibrosis. To try to predict improvements in HbA1C levels over time using all covariates including HCV treatment per se, a logistic regression model was performed (Table 2). The model was statistically significant (*P* < 0.0005) and explained 47% of the variance in HbA1C changes over time, correctly classifying 77% of cases. Treatment with DAAs had a 29.3 times higher odds of improvement in HbA1C levels than nontreatment (95% CI, 8.1 to 106.2; *P* < 0.001). Other statistically significant but less potent predictors of HbA1C improvement included higher baseline serum ALT levels, lower baseline serum AST levels, and older age (Table 3).

In the SVR subgroup (*n* = 55), we examined whether the amount of hepatic fibrosis at the baseline influenced the decrement in blood HbA1C levels following successful treatment. In the 30 patients with either no fibrosis or mild/significant fibrosis (F0-F2) at baseline, the mean blood HbA1C level decreased 1.2 ± 0.3% after a SVR, whereas in the 25 patients with either severe fibrosis or cirrhosis at baseline (F3-F4), mean HbA1C decreased 0.9 ± 0.3% (*P* = 0.48; F0-F2 vs. F3-F4).

## 4. Discussion

HCV infection has a negative impact on glucose metabolism and increases insulin resistance, though the mechanism(s) is (are) unclear. Matsui et al. demonstrated in vitro that hepatitis C viral replication suppresses *GLUT2* expression and hence cellular glucose uptake, doing so by degrading and downregulating hepatocyte nuclear factor 1*α* [16]. Conversely, there has been speculation that insulin resistance and hyperglycemia promote hepatitis C viral replication and are associated with worse clinical outcomes [17, 18].

Observational studies more than a decade ago demonstrated that a SVR achieved with interferon alpha plus ribavirin in patients with chronic hepatitis C could improve glycemic control and insulin sensitivity [19] and possibly even prevent DM [20]. However, these studies were confounded by adverse effects of the medications used to achieve a SVR, including hemolytic anemia from ribavirin which can falsely lower HbA1C levels and interferon-mediated nausea, anorexia, and/or weight loss which can reduce insulin resistance and improve glycemia [21, 22].

A handful of cohort studies have assessed the outcome of treatment of HCV infection using direct acting antiviral drugs (DAAs) on the severity of coexisting diabetes mellitus (DM) [2–6]. In the largest study, Hum et al. studied over 2,000 veterans (98% male) who had type 2 DM and underwent interferon-free and ribavirin-free DAA-based antiviral treatment for their HCV infection [2]. They found that a SVR was associated with improved glycemic control, with decreased HbA1C levels (mean decrease, 1%) and reduced insulin requirements [4]. A significant fall in the blood HbA1C level associated with a SVR was only seen in veterans without severe hepatic fibrosis or cirrhosis [2]. In contrast, in our study with roughly equal proportions of women and men, there were ~1% decrements in mean HbA1C levels in patients with or without severe hepatic fibrosis/cirrhosis.

Our study differs from most of the previous HCV treatment studies in diabetics in that we also compared patients treated with DAAs with untreated HCV-positive controls, whereas earlier studies enrolled only patients treated with DAAs and compared the much larger SVR subgroup with the much smaller non-SVR subgroup [2–6]. The ~1% absolute decrement in blood HbA1C levels with successful HCV treatment 6 months after treatment completion in our study was not seen in untreated controls or in the few patients not achieving a SVR with DAAs. These improvements in glycemic control after SVR could eventually translate into fewer diabetes-related complications. In contrast to improvement in HbA1C with DAA treatment, some authors reported no significant change in the HbA1C level with DAA treatment for Hep C [23, 24, 25]. Chaudhury et al. conducted a prospective study where they showed that achievement of SVR did not lead to improvements in HbA1C [24]. Others reported transient improvement but not long-term glycemic control [26]. The Li et al. study was similar to our study, but they followed up the study population in a longer period and found that the beneficial effect was transient. In contrast, Gilad et al. reported that the beneficial effect was sustained over 1.5 years of follow-up [27]. A couple of studies reported decrease in insulin use after the improvement of HbA1C [2, 4, 5, 28]. In our study, we tried to avoid confounding factors such as diabetic regimen changes which can affect the HbA1C level, and so the diabetes regimen was not changed during the study period. We did not follow all patients after completion of the study period. However, some patients' antidiabetic regimens were later decreased based on their reduced HbA1C levels.

Because the patients with chronic hepatitis C treated with DAAs in our study did not experience any significant changes in body weight or adjustments in their diabetes regimen during and for 6 months after completion of antiviral treatment, it is very likely that the reductions in glycemia (HbA1C levels) that we observed were a consequence of eradication of HCV. Clinicians treating HCV-infected diabetics with DAAs should be aware that glycemia will probably improve and that hypoglycemia might occur, particularly if the patient is receiving insulin, a sulfonylurea, or a meglitinide, requiring careful blood glucose monitoring at home and in the clinic. However, hypoglycemia has not been commonly reported in HCV treatment studies in diabetics on blood glucose-lowering medications and was not a problem observed in our clinic population. We also analyzed the characteristics of those 10 patients with unimproved HbA1C despite a SVR and compared them with SVR patients who improved their HbA1C. We did not find a significant difference in age, viral load, BMI, genotype, or baseline HbA1C in these two subgroups.

In summary, in this controlled study, the single most important factor that predicted improvement in glycemic control in these diabetics with HCV infection was treatment with DAAs. This salutary effect on blood hemoglobin A1C levels was seen in patients with and without advanced fibrosis/cirrhosis and was restricted to patients who achieved an SVR on DAAs.

## Figures and Tables

**Table 1:** Baseline characteristics in the 118 study patients.

	Treated (*n* = 59)	Untreated (*n* = 59)	*P* value
Age (years)^a^	59.2 ± 0.8	53.6 ± 1.1	<0.0001
HbA1C (%)^a^	7.7 ± 0.3	8.2 ± 0.2	NS
Males/females	26/33	25/34	NS
Whites/blacks	19/40	29/30	NS
BMI (kg/m^2^)^a^	30.8 ± 0.8	29.1 ± 0.8	NS
Cirrhosis/no cirrhosis	27/32	26/33	NS
Viral load (copies × 10^6^)^a^	3.8 ± 0.5	2.8 ± 0.4	NS
HCV genotype 1/2/3/4	55/2/1/1	46/10/3/0	0.034^b^
Serum ALT (U/L)^a^	75 ± 6	70 ± 9	NS
Serum AST (U/L)^a^	58 ± 5	61 ± 7	NS
Inflammation: A0-A1/A2-A3^c^	35/24	32/27	NS
Fibrosis: F0-F2/F3-F4^d^	32/27	33/26	NS
Oral medication	13	33	NS
Insulin	6	19	NS
Both	25	7	NS
Diet modification	15	0	—

BMI: body mass index; HbA1C: hemoglobin A1C level (%); HCV: hepatitis C virus; NS: *P* value not statistically significant (*P* > 0.05). ^a^*Mean* ± *SEM*. ^b^Genotype 1 vs. non-1. ^c^Inflammation (A) score on liver biopsy ranging from A0 to A3 (see text). ^d^Fibrosis (F) score on liver biopsy ranging from F0 to F4 (see text).

**Table 2:** Effect of categorical covariates on HbA1C improvement.

Group	*N*	Mean	Std. deviation	*P* value (*t*-test)
Total sample	118	-0.3653	1.342	
Female	67	-0.3507	1.351	NS
Male	51	-0.3843	1.343	
Black	70	-0.5214	1.481	NS
White	48	-0.1375	1.083	
Untreated	59	0.2153	0.361	0.0001^∗^
Treated	59	-0.9458	1.678	

NS: *P* value not statistically significant (*P* > 0.05); *P*: probability.

**Table 3:** Logistic regression model predicting final HbA1C levels in 118 patients with hepatitis C and diabetes mellitus (*n* = 118).

Covariate (1)	Reference variable (0)	Estimate	Chi square	*P* value
Age	—	-0.080	4.490	0.034^∗^
Gender	Female	0.015	0.000	0.975
Race	Black	0.413	0.560	0.453
Viral load	—	-4.9*e*-8	0.460	0.497
Genotype 1	Non-1	-0.104	0.080	0.772
Moderate/severe chronic hepatitis	Minimal/mild chronic hepatitis	0.474	0.960	0.326
Baseline serum ALT level	—	-0.026	7.090	0.008^∗^
Baseline serum AST level	—	0.029	7.190	0.007^∗^
Baseline BMI	—	-0.064	2.280	0.131
Pretreatment HbA1C	—	0.209	1.730	0.189
Severe fibrosis or cirrhosis	No, mild, or significant fibrosis	0.066	0.020	0.889
Treatment with DAAs	No treatment	1.609	25.12	<0.0001^∗^

^∗^ *P* < 0.05. ALT: alanine aminotransferase; AST: aspartate aminotransferase; BMI: body mass index; DAAs: direct acting antiviral agents for HCV; HbA1C: hemoglobin A1C level (%); HCV: hepatitis C virus; *P*: probability.

## Data Availability

The data in tables used to support the findings of this study are included within the article.

## References

[1] Gastaldi G., Goossens N., Clément S., Negro F. (2017). Current level of evidence on causal association between hepatitis C virus and type 2 diabetes: a review. *Journal of Advanced Research*.

[2] Hum J., Jou J. H., Green P. K. (2017). Improvement in glycemic control of type 2 diabetes after successful treatment of hepatitis C virus. *Diabetes Care*.

[3] Abdel Alem S., Elsharkawy A., Fouad R. (2017). Improvement of glycemic state among responders to sofosbuvir-based treatment regimens: single center experience. *Journal of Medical Virology*.

[4] Pavone P., Tieghi T., d’Ettorre G. (2016). Rapid decline of fasting glucose in HCV diabetic patients treated with direct- acting antiviral agents. *Clinical Microbiology and Infection*.

[5] Ciancio A. Q., Bosio R., Bo S. (2018). Significant improvement of glycemic control in diabetic patients with HCV infection responding to direct-acting antiviral agents. *Journal of Medical Virology*.

[6] Fabrizio C., Procopio A., Scudeller L. (2017). HCV and diabetes: towards a ‘sustained’ glycaemic improvement after treatment with DAAs?. *Clinical Microbiology and Infection*.

[7] Pashun R. A., Shen N. T., Jesudian A. (2016). Markedly improved glycemic control in poorly controlled type 2 diabetes following direct acting antiviral treatment of genotype 1 hepatitis C. *Case Reports in Hepatology*.

[8] Drazilova S., Janicko M., Skladany L. (2018). Glucose metabolism changes in patients with chronic hepatitis C treated with direct acting antivirals. *Canadian Journal of Gastroenterology and Hepatology*.

[9] Weidner P., Boettche D., Zimmerer T. (2018). Impact of direct acting antiviral (DAA) treatment on glucose metabolism and reduction of pre-diabetes in patients with chronic hepatitis C. *Journal of Gastrointestinal and Liver Diseases*.

[10] Morales A. L., Junga Z., Singla M. B., Sjogren M., Torres D. (2016). Hepatitis C eradication with sofosbuvir leads to significant metabolic changes. *World Journal of Hepatology*.

[11] Ikeda A., Ikeda K., Takai A. (2017). Hepatitis c treatment with sofosbuvir and ledipasvir accompanied by immediate improvement in hemoglobin a1c. *Digestion*.

[12] The International Expert Committee (2009). International expert committee report on the role of the A1C assay in the diagnosis of diabetes. *Diabetes Care*.

[13] The French METAVIR Cooperative Study Group (1994). Intraobserver and interobserver variations in liver biopsy interpretation in patients with chronic hepatitis C. *Hepatology*.

[14] Bedossa P., Poynard T. (1996). An algorithm for the grading of activity in chronic hepatitis C. *Hepatology*.

[15] Chung R. T., Ghany M. G., Kim A. Y. (2018). Hepatitis C guidance 2018 update: AASLD-IDSA recommendations for testing, managing, and treating hepatitis C virus infection. *Clinical Infectious Diseases*.

[16] Matsui C., Shoji I., Kaneda S., Sianipar I. R., Deng L., Hotta H. (2012). Hepatitis C virus infection suppresses GLUT-2 gene expression via downregulation of hepatocyte nuclear factor 1*α*. *Journal of Virology*.

[17] Negro F., Forton D., Craxì A., Sulkowski M. S., Feld J. J., Manns M. P. (2015). Extrahepatic morbidity and mortality of chronic hepatitis C. *Gastroenterology*.

[18] Negro F. (2014). Facts and fictions of HCV and comorbidities: steatosis, diabetes mellitus, and cardiovascular diseases. *Journal of Hepatology*.

[19] Tahrani A., Bowler L., Singh P., Coates P. (2006). Resolution of diabetes in type 2 diabetic patient treated with IFN-?? and ribavirin for hepatitis C. *European journal of gastroenterology & hepatology*.

[20] Arase Y., Suzuki F., Suzuki Y. (2009). Sustained virological response reduces incidence of onset of type 2 diabetes in chronic hepatitis C. *Hepatology*.

[21] Gross B. N., Cross L. B., Foard J. C., Wood Y. A. (2009). Falsely low hemoglobin A1c levels in a patient receiving ribavirin and peginterferon alfa-2b for hepatitis C. *Pharmacotherapy*.

[22] Greenberg P. D., Rosman A. S., Eldeiry L. S., Naqvi Z., Bräu N. (2006). Decline in haemoglobin A1c values in diabetic patients receiving interferon-alpha and ribavirin for chronic hepatitis C. *Journal of Viral Hepatitis*.

[23] Stine J. G., Wynter J. A., Niccum B., Kelly V., Caldwell S. H., Shah N. L. (2017). Effect of treatment with direct acting antiviral on glycemic control in patients with diabetes mellitus and chronic hepatitis C. *Annals of Hepatology*.

[24] Chaudhury C. S., Sheehan J., Chairez C. (2017). No improvement in hemoglobin a1c following hepatitis c viral clearance in patients with and without HIV. *The Journal of Infectious Diseases*.

[25] Huang J.-F., Huang C.-F., Yeh M.-L. (2017). The outcomes of glucose abnormalities in chronic hepatitis C patients receiving interferon-free direct antiviral agents. *The Kaohsiung Journal of Medical Sciences*.

[26] Li J., Gordon S. C., Rupp L. B. (2019). Sustained virological response does not improve long-term glycaemic control in patients with type 2 diabetes and chronic hepatitis C. *Liver International*.

[27] Gilad A., Fricker Z. P., Hsieh A., Thomas D. D., Zahorian T., Nunes D. P. (2019). Sustained improvement in type 2 diabetes mellitus is common after treatment of hepatitis C virus with direct-acting antiviral therapy. *Journal of Clinical Gastroenterology*.

[28] Dawood A. A., Nooh M. Z., Elgamal A. A. (2017). Factors associated with improved glycemic control by direct-acting antiviral agent treatment in egyptian type 2 diabetes mellitus patients with chronic hepatitis C genotype 4. *Diabetes & Metabolism Journal*.

[29] Mada P. K., Malus M. E., Chen B. (2018). 2222. Impact of sustained virologic response achieved through newer direct acting antivirals in hepatitis C infection on diabetes mellitus. *Open Forum Infectious Diseases*.

